# Ketamine as a prophylactic resilience-enhancing agent

**DOI:** 10.3389/fpsyt.2022.833259

**Published:** 2022-07-28

**Authors:** Audrey G. Evers, James W. Murrough, Dennis S. Charney, Sara Costi

**Affiliations:** ^1^Depression and Anxiety Center for Discovery and Treatment, Department of Psychiatry, Icahn School of Medicine at Mount Sinai, New York, NY, United States; ^2^Department of Neuroscience, Icahn School of Medicine at Mount Sinai, New York, NY, United States; ^3^Department of Pharmacology and Systems Therapeutics, Icahn School of Medicine at Mount Sinai, New York, NY, United States; ^4^Psychopharmacology and Emotion Research Laboratory (PERL), Department of Psychiatry, Warneford Hospital, University of Oxford, Oxford, United Kingdom; ^5^Oxford Health National Health Service (NHS) Foundation Trust, Warneford Hospital, Oxford, United Kingdom

**Keywords:** ketamine, prophylactic, resilience, stress, prevention

## Abstract

Stress exposure is one of the greatest risk factors for psychiatric illnesses, including major depressive disorder (MDD) and posttraumatic stress disorder (PTSD). Enhancing stress resilience could potentially protect against the development of stress-induced psychiatric disorders, yet no resilience-enhancing pharmaceuticals have been developed to date. This review serves to consider the existing evidence for a potential pro-resilience effect of ketamine in rodents as well as the preliminary evidence of ketamine as a prophylactic treatment for postpartum depression (PPD) in humans. Several animal studies have demonstrated that ketamine administered 1 week prior to a stressor (e.g., chronic social defeat and learned helplessness) may protect against depressive-like behavior. A similar protective effect has been demonstrated against PTSD-like behavior following Contextual Fear Conditioning (CFC). Recent work has sought to explore if the administration of ketamine prevented the development of postpartum depression (PPD) in humans. Researchers administered ketamine immediately following caesarian-section and found a significantly reduced prevalence of PPD in the ketamine-treated groups compared to the control groups. Utilizing ketamine as a resilience-enhancing treatment may have unique applications, including leading to a deeper understanding of the neurobiological mechanism underlying resilience. Future trials aiming to translate and replicate these findings with humans are warranted.

## Introduction

Stress exposure is one of the greatest risk factors for psychiatric illnesses, including major depressive disorder (MDD) and posttraumatic stress disorder (PTSD). MDD is a pervasive condition affecting more than 300 million people and is a leading cause of disability worldwide (1). Currently available treatments for MDD and PTSD include lifestyle changes, psychological therapies, antidepressants, and other medications. The glutamate N-methyl-D-aspartate (NMDA) receptor antagonist ketamine has been associated with rapid antidepressant and anti-suicidal effects in several clinical trials on patients with treatment-resistant depression [TRD; (2–5)] and also appears to be effective for the treatment of PTSD (6, 7). Ketamine trials with PTSD have found improved overall PTSD symptom severity and specifically improved symptom clusters of intrusion, avoidance, and negative mood and cognitions. In 2019, esketamine nasal spray (SPRAVATO) was FDA approved for the treatment of Treatment Resistant MDD (TRD) and in 2020, the label was updated to include MDD with suicidal ideation or behavior (8). Due to its novel mechanism of action, ketamine's use as an antidepressant is considered one of the biggest developments in psychiatry and psychopharmacology in decades (9).

Enhancing stress resilience in at-risk populations could potentially protect against the development of stress-induced psychiatric disorders, yet no resilience-enhancing pharmaceuticals have been identified to date. Stress resilience is a multidimensional construct with a variety of definitions. Stress resilience has been defined as the ability to experience stress without developing psychopathology but has also been associated with the concept of adaptation (or the ability to “bounce back”), the ability to quickly recover after a stressor, or the capacity to maintain functioning following adversity (40). In recent years, there is emerging evidence from preclinical data that the administration of ketamine prior to an acute stressor prevents the development of depressive-like or PTSD-like behavior in animals (see Table 1). In addition, recent human clinical trials suggest that the administration of ketamine immediately following cesarean section may reduce the incidence of postpartum depression (PPD). These results are reported in Table 2. This manuscript serves to summarize the current evidence on the potential of ketamine as a protective agent for the development of stress-related disorders. If confirmed by clinical trials in humans, this may represent a significant paradigm shift in the prevention of disorders such as MDD, PTSD, and PPD and provide a novel understanding of the neurobiological mechanism of resilience.

**Table 1:** The prophylactic effect of ketamine in rodent models on behavioral outcomes.

**Paper**	**Drug and dose**	**Animal used**	**Stress paradigm**	**Outcome**	**Time**	**Behavioral results**
Ketamine as a prophylactic against stress-induced depressive-like behavior (11)	Ketamine 10, 30, and 90 mg/kg	Male 129S6/SvEvTac mice (8–10 weeks old)	SD (2 weeks)	FST +1 day, DI +2 days, EPM +4 days	1 week between dose and stress	Ketamine (30 mg/kg, but not 10 or 90 mg/kg) significantly decreased behavioral despair (immobility time in the FST) following the SD when compared with saline. SD-ketamine mice exhibited a significantly increased willingness to interact with the dominant mouse when compared with SD-Sal mice. No effect on EPM: unclear if ketamine is an anxiolytic.
	Ketamine 30 mg/kg	Male 129S6/SvEvTac mice (8–10 weeks old)	LH (2 weeks)	LH Testing (Shock Escape Protocol)	1 week between dose and stress	Mice injected with ketamine (30 mg/kg) had a decreased latency to escape and the session length was significantly shorter in the ketamine group. Ketamine protection may not be limited to just SD stress.
	Ketamine 10, 30, and 90 mg/kg	Male C57BL/6NTac mice (8 weeks old)	21-day CORT	FST, ST, NSF	1 week between dose and stress	Ketamine (90 mg/kg, but not 10 or 30 mg/kg) was the most effective at preventing the chronic CORT-induced depressive-like phenotype and ketamine treated-mice showed significantly decreased immobility time on the FST, demonstrating the protective effect of ketamine at a higher dose.
Previous ketamine produces an enduring blockade of neurochemical and behavioral effects of uncontrollable stress (13)	Ketamine 10 mg/kg	Male Sprague Dawley rats (64–69 days old)	IS	JSI 24 h post-IS	2 h, 1 week, or 2 weeks between dose and stress	Ketamine (10 mg/kg) blocked behavioral effects of IS at all intervals (2 h, 1 week, and 2 weeks) at *p* < 0.01 compared to saline.
Prophylactic ketamine attenuates learned fear (17)	Ketamine 30 mg/kg	Male 129S6/SvEvTac mice (8 weeks old)	3-shock CFC	E and R	24 h, 1 week, or 1 month between dose and stress	Ketamine (30 mg/kg) treated mice exhibited significantly less freezing behavior during extinction exposure 5 days after CFC: ketamine attenuated fear response 1 week before CFC, but not 1 month or 24 h.
Prophylactic ketamine alters nucleotide and neurotransmitter metabolism in brain and plasma following stress (18)	Ketamine 30 mg/kg	Male 129S6/SvEvTac mice (8 weeks old)	3-shock CFC	Context Re-exposure	1 week between dose and stress	Ketamine (30 mg/kg) compared to saline attenuated fear response during context re-exposure.
Ventral CA3 activation mediates prophylactic ketamine efficacy against stress-induced depressive-like behavior (12)	Ketamine 30 mg/kg	Male 129S6/SvEvTac mice (8 weeks old)	SD and 3-shock CFC	FST	1 week between dose and stress	Mice treated with ketamine (30 mg/kg) exhibited attenuated fear compared with saline treated mice. On day 2 of the FST (6 days after CFC), ketamine-treated (30 mg/kg) mice exhibited decreased immobility compared with saline.
Prophylactic ketamine treatment promotes resilience to chronic stress and accelerates recovery: correlation with changes in synaptic plasticity in the CA3 subregion of the hippocampus (14)	Ketamine 3 mg/kg	Male C57BL6J mice (3 months old)	CUS	FST 2 days after CUS and 8-day SPT	1 h between dose and stress	More anhedonic mice (based on sucrose preference over water) in saline group (60%) compared to ketamine group (37%) 24 h after CUS. Ketamine administration improved anhedonia recovery during an 8-day recovery period (*p* = 0.022). No difference between ketamine and saline groups on FST.
Sex-specific neurobiological actions of prophylactic (R,S)-ketamine, (2R,6R)-hydroxynorketamine, and (2S,6S)-hydroxynorketamine (23)	(R,S)-ketamine, (2R,6R)-HNK, (2S,6S)-HNK	Male 129S6/SvEv mice (8 weeks old)	3-shock CFC	FST	1 week between dose and stress	(R,S)-ketamine (30 mg/kg) and (2S,6S)-HNK (0.025, 0.075, 0.1, 0.3, 10, and 30 mg/kg), but not (2R,6R)-HNK attenuated learned fear on day 1 of the FST. On day 2, (R,S)-ketamine (30 mg/kg) and (2R,6R)-HNK (0.075 mg/kg), but not (2S,6S)-HNK, reduced depressive-like behavior compared to saline.
	(R,S)-ketamine, (2R,6R)-HNK, (2S,6S)-HNK	Female 129S6/SvEv mice (8 weeks old)	3-shock CFC	FST	1 week between dose and stress	(R,S)-ketamine and metabolites do not attenuate learned fear in female mice. On FST day 2, (R,S)-ketamine (10 mg/kg) and (2R,6R)-HNK (0.025 mg/kg) significantly reduced depressive-like behavior compared to saline.
	(R,S)-ketamine, (2R,6R)-HNK	Female 129S6/SvEv mice (8 weeks old) OVX and sham	CFC	FST	1 week between dose and stress	Ovarian hormones mediate the prophylactic effect of (R,S)-ketamine and (2R,6R)-HNK in female mice as the drugs did not affect immobility time during the FST in the OVX groups.
	(R,S)-ketamine, (2R,6R)-HNK, (2S,6S)-HNK	Female 129S6/SvEv mice (8 weeks old)	LH	FST and EPM	1 week between dose and stress	(R,S)-ketamine and (2R,6R)-HNK prevent LH induced depressive-like behavior in female mice. Both drugs significantly reduced immobility time in FST. Unlike in male mice, (R,S)-ketamine and (2R,6R)-HNK do not alter helplessness behavior in female mice.
Sex differences in the sustained effects of ketamine on resilience to chronic stress (22)	Ketamine 10 mg/kg	Male and Female C57Bl/6 mice (8 weeks old)	UCMS (4 week)	FST	1 week between dose and stress	Ketamine (10 mg/kg) administered prior to 4 weeks of UCMS significantly reduced immobility time on the FST in male mice, but not female mice.
Ketamine, but not guanosine, as a prophylactic agent against corticosterone-induced depressive-like behavior: possible role of long-lasting pro-synaptogenic signaling pathway (15)	Ketamine (1 or 5 mg/kg) or Guanosine (1 or 5 mg/kg)	Male C57BL/6NTac Swiss mice (55–60 days old)	21 day CORT (20 mg/kg)	TST and SPT	1 week between dose and stress	Ketamine (5 mg/kg, but not 1 mg/kg) significantly prevented increase in immobility time, grooming latency, and reduced total time grooming (*p* < 0.01) compared to saline. Unlike ketamine, guanosine did not prevent any behavioral changes induced by CORT administration.
Inhibition of a descending prefrontal circuit prevents ketamine-induced stress resilience in females (21)	Ketamine 10 mg/kg, 40 mg/kg	Female Sprague Dawley rats (57–70 days old)	IS	JSE +24 h from IS	1 week between dose and stress	Ketamine (10 mg/kg, but not 40 mg/kg) given 1 week prior to IS completely blocked the effect of IS on JSE in female rats.

*FST, Forced Swim Test; LH, Learned Helplessness; CFC, Contextual Fear Conditioning; CUS, Chronic Unpredictability Stress; E, Extinction; R, Reinstatement; DI, Dominant Interaction; EMP, Elevated Maze Plus; TST, Tail Suspension Test; SPT, Splash Test; SD, Chronic Social Defeat; CORT, Chronic Corticosterone; SPT, Sucrose Preference Test; IS, Inescapable Shock; JSI, Juvenile Social Investigation; OVX, Ovariectomized; ST, Sucrose Splash Test; NSF, Novelty Suppressed Feeding; UCMS, Unpredictable Chronic Mild Stress; JSE, Juvenile Social Exploration*.

**Table 2:** Effect of ketamine on prevention postpartum depression (PPD).

**Paper**	**Drug and dose**	**Sample**	**Control used**	**Outcome measure**	**Primary outcome**	**Secondary outcomes**	**Exploratory findings**
Prophylactic use of ketamine reduces postpartum depression in Chinese women undergoing cesarean section (27)	Ketamine 0.5 mg/kg +10 minutes after c-section + PCIA device: sufentanil (100 ug), ketamine (160mg), and palonosetron hydrochloride (0.25 mg)	*n* = 654 (*n* = 343 in each group)	0.9% saline + PCIA device: sufentanil (100 ug) and palonosetron hydrochloride (0.25 mg)	EPDS +1 day, +2 days, +4–6 days (Secondary Outcome), and +6–8 weeks (Primary Outcome) post c-section	PPD prevalence in ketamine group significantly lower (12.8%) than control group (19.6%) (*p* = 0.02) at 6–8 weeks post c-section.	EPDS score at days 4–6 was significantly lower in the ketamine group (*p* = 0.007). Prevalence of postpartum blues significantly lower in ketamine group (11.9%) than control (18.3%) (*p* = 0.022). Reduction in suicidal ideation was significantly higher in the ketamine group (*p* = 0.017).	Stronger effect in those with a history of moderate stress during pregnancy (*p* = 0.003), antenatal depressive symptoms (*p* = 0.05) and antenatal suicidal ideation (*p* = 0.02).
The effect of ketamine on preventing postpartum depression (30)	Ketamine 0.5 mg/kg + Nesdonal 1–2 mg/kg	*n* = 134 (*n* = 67 in each group)	Nesdonal 3–5 mg/kg of body weight intravenously injected during induction of anesthesia	EPDS +2 weeks and +4 weeks post c-section	PPD prevalence in ketamine group was significantly lower than control (*p* < 0.001) at 4 weeks postpartum. The mean EPDS score in the ketamine group (10.84) was significantly less than the control group (13.09; *p* < 0.001) at 4 weeks postpartum.	PPD prevalence in ketamine group significantly lower than control (*p* < 0.001) at 2 weeks postpartum. EPDS score in the ketamine group (11.82) was significantly less than the control group (14.34; *p* < 0.001) at 2 weeks postpartum.	In the control group, the mean EPDS score was increased 2-weeks after the c- section compared to before and the mean EPDS score was significantly decreased 4 weeks after the c-section in comparison to two weeks after (*p* < 0.001).
S-ketamine as an adjuvant in patient controlled intravenous analgesia for preventing postpartum depression: a randomized controlled trial (31)	PCIA device containing: S-ketamine (0.5 mg/kg), sufentanil (2 μg/kg), and tropisetron (10 mg)	*n* = 380 (*n* = 190 in each group)	PCIA device containing: sufentanil (2 μg/kg), and tropisetron (10 mg)	Incidence of PPD, EPDS scores before surgery, +3, +14, and +28	PPD prevalence in the S-ketamine group was significantly lower than the control group (*p* < 0.05) at 3 days and 14 days post-c-section (17.6 and 8.2% vs. 24.2 and 9.8%)	EPDS score intergroup differences were significant at 3 days (*p* < 0.001) and 14 days (*p* < 0.001) postpartum.	VAS scores were significantly lower in the S-ketamine group at 4, 8, 12, and 24 h post-c-section.

*PCIA, Patient Controlled Intravenous Analgesia Device; EPDS, Edinburgh Postnatal Depression Scale; PPD, Postpartum Depression*.

## Ketamine as a prophylactic agent for depressive-like behavior

Several models have reliably induced pro-depressive behaviors in rodents, including Chronic Social Defeat (SD), Learned Helplessness (LH), Inescapable Shock (IS), Chronic Unpredictability Stress (CUS), and chronic corticosterone (CORT). In recent years, several authors have demonstrated a single administration of ketamine exerts a protective effect against the depressive-like behavior induced by these models. In 2016, Brachman et al. treated male mice with ketamine (10, 30, or 90 mg/kg) or saline 1 week prior to a 2-week SD and assessed behavior using the Forced Swim Test (FST) 1 day post-SD along with the Dominant Interaction (DI) social interaction test 2 days post-SD. The FST is commonly used to assess depressive-like behavior: immobility time during the FST has been used as a marker of negative mood, hopelessness, or despair (10). Similarly, DI is a robust way to test the induction of depressive-like behavior by SD: time spent investigating an empty enclosure (increased time indicates a depressive-like behavior) and willingness to interact with the dominant mouse (reduced willingness indicates depressive-like behavior) can be measured. Ketamine-treated mice (30 mg/kg, but not 10 or 90 mg/kg) showed significantly reduced immobility time during the FST and significantly increased time exploring a social target mouse compared to saline-treated mice during the DI, consistent with a reduction in the pro-depressive effects of SD and enhancement of stress resilience (11). In 2018, Mastrodonato et al. replicated these results after treating male mice with ketamine (30 mg/kg) 1 week prior to SD followed by the FST. Mice treated with ketamine exhibited significantly less immobility time compared with saline-treated mice on day 2 of the FST (12). Furthermore, Amat et al. (13) administered ketamine 10 mg/kg to male rats at varying times (2 h, 1 week, and 2 weeks) before IS, a laboratory inducing anxiety procedure in rodents. Ketamine blocked the behavioral impairment of IS at all time intervals compared to saline-treated rats on the Juvenile Social Interaction (JSI), which has been used to test social interest and motivation in rats (13).

In another experiment, Brachman et al. (11) treated male mice with ketamine (30 mg/kg) 1 week prior to a 2-week LH protocol where mice were delivered repeated, inescapable shocks. Ketamine-treated mice had a decreased latency to escape the shock escape protocol during LH testing and the session length was significantly shorter in the ketamine-treated group, suggesting a blunting of the depressive effect of LH (11).

Anhedonia, a common symptom of depression, is characterized by a reduced ability to feel pleasure. CUS has been shown to cause anhedonia-like behavior in rodent models as measured by the Sucrose Preference Test (SPT). Krzystyniak et al. (14) administered ketamine (3 mg/kg) or saline to male mice 1 h before the CUS protocol, followed by an 8-day SPT, except on day 2, when the FST was administered. They reported an increase in anhedonic behaviors in mice treated with saline (60%) compared to the ones treated with ketamine (37%), as quantified by an impaired sucrose preference over water 24 h after the CUS protocol. They also found that ketamine (3 mg/kg) administration improved anhedonia recovery during the 8-day SPT in ketamine-treated mice exhibiting anhedonia-like behavior. However, inconsistent with previously mentioned findings, no difference between groups in immobility time during the FST was reported (14). Of note, the different dosage of ketamine, 3 mg/kg rather than 30 mg/kg used in previous findings (11, 12) as well as the different timing employed (2 days post ketamine rather than 1 week), may suggest that the prophylactic effect of ketamine against depressive-like symptoms is dose and time-dependent.

Brachman et al. (11) also tested the effect of ketamine (90 mg/kg) on male mice prior to a 21-day CORT treatment. CORT administration has been shown to induce depressive-like behavior in mice as measured with the Tail Suspension Test (TST), Splash Test (SPT), and FST (11, 15). Ketamine (90 mg/kg) prevented the chronic CORT-induced depressive-like phenotype and treated mice showed significantly decreased immobility time on the FST, demonstrating the protective effect of ketamine also at a higher dose (11). Similarly, Camargo et al. (15) treated male mice with ketamine (5 mg/kg or 1 mg/kg) 1 week prior to 21-day CORT (20 mg/kg) treatment. Ketamine-treated (5 mg/kg, but not 1 mg/kg) mice showed significantly less immobility time on the TST, grooming latency, and reduced total time grooming on the SPT. Grooming during the Splash Test is considered a self-care behavior and a marker of anhedonia in mice (15). To investigate whether ketamine exerted a similar effect when administered after stress rather than before, ketamine (30 mg/kg) was administered 1 day after a 28-day CORT treatment. However, post-stress treatment with ketamine did not affect immobility time in the FST (11).

## Ketamine as a prophylactic agent for PTSD-like behavior

Contextual Fear Conditioning (CFC) has reliably shown to produce PTSD- and depressive-like behavior in mice. The CFC utilizes shocks, most commonly one or three shocks, context re-exposure (R) and extinction (E) to assess the ability of mice to learn and associate environmental cues and aversive experiences. PTSD is characterized by the persistence of intrusive and debilitating traumatic memories (16). In rodents, PTSD-like behavior is associated with learned fear when re-exposed to aversive contexts. During re-exposure tests, freezing behavior is often measured as an index of fear memory. McGowan et al. (17) treated mice with 30 mg/kg of ketamine or saline at different time points (24 h, 1 week, or 1 month) before a 3-shock CFC protocol. Mice treated with ketamine (30 mg/kg) 1 week prior to CFC showed significantly less freezing behavior during extinction exposure 5 days after CFC, while mice treated 24 h or 1 month before CFC did not differ from the control group (17). Of note, ketamine administered after the CFC at various time points did not alter fear expression (17). Interestingly, there was no effect of ketamine on fear expression or depressive-like behavior in the FST when administered 1 week before a “stronger” re-exposure. This may suggest that ketamine's prophylactic effect is dependent on the severity of the stressor (17). McGowan et al. (18) replicated these findings while investigating markers associated with the prophylactic effect of ketamine. Ketamine appeared to significantly alter metabolites in the prefrontal cortex (PFC) and in the hippocampus (HPC) following CFC. Additionally, amino acid derived neurotransmitters and precursors significantly changed following ketamine and stress in PFC and HPC tissue and plasma. These changes were durable (detectable at 2 weeks post ketamine administration) and observable in the PFC, HPC, and plasma. Interestingly, these alterations were not found in ketamine-treated mice that did not undergo the CFC stress, suggesting that the interaction between stress and ketamine may be key to creating long-lasting metabolic changes that affect stress-related behavior (18).

In 2018, Mastrodonato et al. treated mice with ketamine (30 mg/kg) or saline 1 week prior to a 3-shock CFC protocol and showed that mice treated with ketamine exhibited significantly attenuated fear compared to saline-treated mice on day 2 of the FST, suggesting that the prophylactic effect ketamine can be extended also to the PTSD-like behavior induced by CFC (12). Furthermore, the authors sought to explore the neural mechanisms associated with the pro-resilient effect of ketamine. Through the use of viral vectors, the authors were able to test the hypothesis that the mice that received ketamine prior to a stressor (CFC) showed an alteration of stress-related memories (CFC-related) in the hippocampus (12). Overall, these findings suggest that ketamine may impact the encoding of negative memories. While ketamine has been studied in cue-specific fear conditioning models (19, 20) wherein it appeared to disrupt contextual fear reconsolidation, it has not yet been studied when administered prophylactically prior to CFC.

## Ketamine as a prophylactic agent for anxiety-like behavior

The Elevated Plus Maze (EPM) test is widely used to assess anxiety-related behavior in rodents. In the previously discussed study from Brachman et al. (11), the EPM was conducted 4 days after SD and no effect of ketamine was found on the EPM results. Dolzani et al. (21) sought to replicate the findings of Amat et al. (13) in a population of female rats. Ketamine (10 mg/kg) was administered 1 week prior to IS and the effect of ketamine was assessed 24 h after on the Juvenile Social Exploration (JSE) test, which is a measure of anxiety-like behavior. Another group was given 40 mg/kg (high dose) of ketamine. Low-dose ketamine (10 mg/kg) given 1 week prior to IS completely blocked the effect of IS on JSE, but high-dose ketamine (40 mg/kg) behaved the same as control. It remains unclear if ketamine prevents the development of anxiety-related behaviors in mice, but it appears it could be dose-dependent.

## Generalizability of prophylactic effect of ketamine: Is it the same for female rodents?

Along with Dolzani et al. (21), other researchers have sought to test if the prophylactic effects of ketamine and its metabolites (2R,6R)-hydroxynorketamine and (2S,6S)-hydroxynorketamine extend to female animals and to explore the potential contribution of sex hormones. Okine et al. (22) administered ketamine (10 mg/kg) to male and female mice 1 week prior to a 4-week Unpredictable Chronic Mild Stress (UCMS) protocol. They tested the effect of the UCMS with the FST and found that ketamine promoted resilience in male mice, but not in female mice. More recently, Chen et al. (23) used the LH protocol and three-shock CFC to test the prophylactic effect of ketamine in female rodents. In this work, ketamine and (2R,6R)-HNK prevented LH-induced depressive-like behavior in female mice along with reducing immobility time during the FST, suggesting that the stress resilience enhancement of ketamine applies to both male and female mice. Furthermore, ketamine (30 mg/kg) and (2R,6R)-HNK (0.075 mg/kg), but not (2S,6S)-HNK, administered 1 week prior to a three-shock CFC reduced depressive-like behavior in male mice on day 2 of the FST. Similarly, ketamine (10 mg/kg), (2R,6R)-HNK (0.025 mg/kg), and (2R,6R)-HNK (0.025 mg/kg) administered 1 week prior to the three-shock CFC significantly reduced depressive-like behavior in female mice on day 2 of the FST, providing evidence for a protective effect of ketamine following an acute stressor also on female rodents. Both ketamine (30 mg/kg) and (2S,6S)-HNK at a variety of doses, but not (2R,6R)-HNK, when administered 1 week prior to a three-shock CFC induced attenuated learned fear on day 1 of the FST. Unlike male mice, ketamine and its metabolites did not attenuate learned fear in female mice. To further explore the timing of administration in the stress resilience model, ketamine and its metabolites were administered 3 days or 24 h prior to the CFC rather than 1 week. (2R,6R)-HNK, but not ketamine, was prophylactic when administered 3 days, but not 24 h, before stress in female mice, suggesting a prominent role of the timing of ketamine administration in mediating the stress resilience effect. Although it is possible that the lower dose of ketamine in Okine et al. (10 mg/kg rather than 30 mg/kg used by Chen et al.) may explain some of the variations in the prophylactic effect of ketamine, the lack of available data on higher dose of ketamine using the UCMS protocol limits the possibility to draw definite conclusions.

## Ketamine as a prophylactic agent for postpartum depression

Recent studies have explored the potential of ketamine for the prevention of postpartum depression (PPD) in humans. Despite not representing translational trials of the aforementioned animal models, these studies also investigated the effect of ketamine ahead of the development of psychopathology and specifically tested if ketamine exerts a protective effect against the development of mood and anxiety disorders following childbirth. PPD affects an estimated 10–20% of mothers (24, 25) and unique challenges face providers in the treatment of PPD, including the potential exposure of the newborn to medications during pregnancy and/or breastfeeding. Currently, only one medication, brexanolone, is available and received Food and Drug Administration approval for the treatment of PPD (26).

Ma et al. (27) reported the results of 654 women undergoing cesarean section (c-section) that were randomized to either 0.5 mg/kg of ketamine or saline *via* epidural bolus 10 min after c-section. Following initial administration of ketamine or saline, participants were provided with a Patient Controlled Intravenous Analgesia (PCIA) device. Patients undergoing c-section were enrolled because the procedure involves the use of anesthesia regardless of study participation. Further, ketamine has been commonly used as a general anesthetic in patients undergoing planned c-section since its safety profile in pregnant patients is well-established (28). Participants that were randomized to ketamine received a PCIA device of sufentanil (100 μg), palonosetron hydrochloride (0.25 mg), and ketamine (160 mg). Control subjects were provided the same PCIA device without the addition of ketamine. Participants were assessed with the Edinburgh Postnatal Depression Scale [EPDS; (29)] at various time points postpartum. The primary outcome at 6–8 weeks postpartum showed a significantly lower prevalence of PPD in the ketamine group (12.8%) than in the control group (19.6%). The secondary outcome on days 4–6 postpartum showed a significantly lower mean EPDS score and lower prevalence of postpartum blues in the ketamine group (11.9%) than in the control group (18.3%). Notably, a reduction in suicidal ideation 4–6 days postpartum was also significantly higher in the ketamine group compared to the control group. Finally, the effect of ketamine appeared more pronounced in women with a history of moderate stress during pregnancy, antenatal depressive symptoms, and suicidal ideation.

A similar study by Alipoor et al. (30) was conducted on 134 women undergoing c-section randomized to either ketamine (0.5 mg/kg) plus nesdonal (1–2 mg/kg) or nesdonal (3–5 mg/kg) alone administered intravenously during the induction phase of anesthesia. Participants were assessed with the EPDS at 2 and 4 weeks postpartum. The primary outcome at 4 weeks postpartum showed that PPD prevalence (EPDS score > 9) in the ketamine group was significantly lower compared to the control group and that the mean EPDS score in the ketamine group (10.84) was significantly lower than the control group (12.09). The secondary outcome at 2 weeks postpartum showed a significantly lower prevalence of PPD in the ketamine group and a mean EPDS in the ketamine group (11.82) significantly lower than the control group (14.34). A third randomized trial from Han et al. (31) explored the effect of a PCIA device with S-ketamine (0.5 mg/kg) given to patients immediately following c-section delivery. Patients randomized to receive the PCIA device with S-ketamine had significantly less prevalence of PPD at 3 and 14 days postpartum. However, these findings must be interpreted with caution, as the results reported reflect the mean difference and prevalence difference between groups drawn from uncorrected *t*-tests at the pre-determined time points.

## Discussion

Current evidence suggests a potential for ketamine as a pharmacological agent for the prevention of stress-related behavior in animal models of stress-related disorders and recent findings from Ma et al. (27) and Alipoor et al. (30) provide encouraging evidence that this prophylactic effect may apply to humans. Ketamine as a treatment for psychiatric disorders was discovered as early as 2000 and evidence has grown over the years to support its treatment efficacy for MDD, TRD, suicidality, and PTSD (2–6, 32). However, there has not yet been any study published testing the potentially pro-resilient effect of ketamine in humans with the exception of studies on postpartum depression.

Current evidence-based methods for enhancing resilience are limited to therapeutic lifestyle changes (TLC) focused on smoking habits, alcohol use, diet, physical exercise, obesity, and stress management (33). Successful lifestyle changes are often dependent on psychotherapy and motivational interviewing, which can be expensive and inaccessible (34). While treating the population with ketamine to prevent psychopathology is unrealistic, the implementation of ketamine as a prophylactic treatment may have a potential application for individuals who are at risk of being exposed to a high level of stress within a specific time frame, such as soldiers prior to military deployment or first responders. Interestingly, the preclinical data discussed in the current review (17, 23) suggests that the timing of ketamine administration plays a key role in its stress resilience action and further research is required to optimize administration timing in humans. More broadly, however, the results of these data may inform about the neurobiological mechanisms underpinning resilience. Current practice in psychiatry is focused on the treatment of symptoms, while resilience is generally conceptualized as the absence of disease. Considering resilience as an active state rather than simply as the absence of illness may allow a more in-depth understanding of the neurobiological mechanisms involved, leading to a deeper understanding of resilience-related neural mechanisms and ultimately to the identification of novel target for the prevention of stress-related disorders. Evidence from rodent models discussed in this review may point to the glutamatergic system, the PFC, and the HPC as potential mediators of resilience.

These findings are seemingly consistent with previous work underscoring the neurobiological underpinnings of resilience. In a 2019 review, Cathomas et al. (41) described resilience as an active mechanism rooted in the PFC, HPC, locus coeruleus (LC), ventral tegmental area (VTA), and nucleus accumbens (NAc) as well as peripherally in the body in the innate and adaptive immune systems and the gut microbiota. Consistent with McGowan et al. (18) and Mastrodonato et al. (12), the PFC and HPC are key regions for processing stress and for resilience. Of note, the HPC is highly reactive to hypothalamic-pituitary-adrenal (HPA) axis activation and hippocampal neurogenesis appears foundational to a resilient phenotype (42, 43). However, Brachman et al. (11) found no effect of prophylactic ketamine on hippocampus neurogenesis, and similarly later work from the same animal cohort (35) found no effect of prophylactic ketamine on neurogenesis. At the current time, the neurobiological mechanism underpinning the prophylactic effect of ketamine remains largely unknown. However, the PFC and HPC are also relevant regions within the mesolimbic dopamine pathway, part of a discrete reward circuit, where dopamine neurons projections from the VTA reach the NAc, HPC, and PFC. Relevantly, resilient mice display normal projections of dopamine neurons while susceptible mice display hyperactivity of dopamine neurons (36). The NAc also integrates glutamatergic inputs from the HPC, PFC, and other brain regions—further supporting the hypothesis that resilience may be related to the modulation of glutamatergic transmission (37). Further, psychosocial stress has also repeatedly been shown to be associated with a pro-inflammatory status and current evidence suggests that ketamine may reduce levels of pro-inflammatory cytokines (38, 39). Whether this plays a role in the pro-resilient effect of ketamine is yet to be determined.

This does not serve as a comprehensive review and is primarily focused on the behavioral outcomes showing a pro-resilient effect of ketamine. The implementation of ketamine as a resilience-enhancing agent represents a novel approach to the treatment and prevention of stress-related psychiatric disorders such as MDD or PTSD. The preclinical data discussed in this review suggests that ketamine and its metabolites can be effective at preventing depression, PTSD, and possibly anxiety-like behaviors in animals. In humans, initial evidence from the use of ketamine for the prevention of postpartum depression appears encouraging. Further trials aiming at exploring if the potential stress-preventative effect of ketamine applies also to humans are warranted. Future studies aiming to translate these results into humans should consider the different doses of ketamine and its metabolites used in these rodent models. Although a dose between 10 and 30 mg/kg administered intraperitoneally has been widely accepted as a translational animal model to study the antidepressant effect of ketamine, a variety of factors like the route of administration, metabolism of the drug, and side effect profiles should be accounted for. Substantial uncertainty remains around a standardized prophylactic ketamine protocol including timing, mouse strain, and the optimal prophylactic dosing for animal models and for humans.

## Author contributions

All authors devised the article. SC, AE, and JM conceived the paper. AE wrote the first draft of the article and drafted the tables. All authors commented on drafts of the article. All authors contributed to the article and approved the submitted version.

## Funding

This study was funded by the Ehrenkranz Laboratory for the study of Human Resilience and by a generous gift from the Gottesman Foundation. SC was funded by a Wellcome Trust Clinical Doctoral Research Fellowship.

## Conflict of interest

DC (Dean of Icahn School of Medicine at Mount Sinai) is a named co-inventor on several issued U.S. patents and several pending U.S. patent applications filed by the Icahn School of Medicine at Mount Sinai (ISMMS) related to ketamine and esketamine for treatment-resistant depression, suicidal ideation, and other disorders. ISMMS has entered into a licensing agreement with Janssen Pharmaceuticals, Inc. and it has and will receive payments from Janssen under the license agreement related to these patents. As a co-inventor, DC is entitled to a portion of the payments received by the ISMMS. Since SPRAVATO (esketamine) has received regulatory approval for treatment-resistant depression, ISMMS and DC as its employee and a co-inventor, will be entitled to additional payments, under the license agreement. SC has provided consultation services for Guidepoint and TCG Crossover. In the past 5 years, JM has provided consultation services and/or served on advisory boards for Allergan, Boehreinger Ingelheim, Clexio Biosciences, Fortress Biotech, FSV7, Global Medical Education (GME), Otsuka, and Sage Therapeutics. JM is named on a patent pending for neuropeptide Y as a treatment for mood and anxiety disorders and on a patent pending for the use of ezogabine and other KCNQ channel openers to treat depression and related conditions.

## Publisher's note

All claims expressed in this article are solely those of the authors and do not necessarily represent those of their affiliated organizations, or those of the publisher, the editors and the reviewers. Any product that may be evaluated in this article, or claim that may be made by its manufacturer, is not guaranteed or endorsed by the publisher.
